# CT Imaging of Coronary Stents: Past, Present, and Future

**DOI:** 10.5402/2012/139823

**Published:** 2012-09-11

**Authors:** Andreas H. Mahnken

**Affiliations:** Department of Diagnostic and Interventional Radiology, University Hospital, RWTH Aachen University, Pauwelsstrasse 30, 52074 Aachen, Germany

## Abstract

Coronary stenting became a mainstay in coronary revascularization therapy. Despite tremendous advances in therapy, in-stent restenosis (ISR) remains a key problem after coronary stenting. Coronary CT angiography evolved as a valuable tool in the diagnostic workup of patients after coronary revascularization therapy. It has a negative predictive value in the range of 98% for ruling out significant ISR. As CT imaging of coronary stents depends on patient and stent characteristics, patient selection is crucial for success. Ideal candidates have stents with a diameter of 3 mm and more. Nevertheless, even with most recent CT scanners, about 8% of stents are not accessible mostly due to blooming or motion artifacts. While the diagnosis of ISR is currently based on the visual assessment of the stent lumen, functional information on the hemodynamic significance of in-stent stenosis became available with the most recent generation of dual source CT scanners. This paper provides a comprehensive overview on previous developments, current techniques, and clinical evidence for cardiac CT in patients with coronary artery stents.

## 1. Rationale for CT Imaging of Coronary Stents

Coronary artery stenting was pioneered in the mid 1980s [1]. It rapidly replaced “plain old balloon angioplasty” for coronary revascularization and became the most commonly used revascularization technique in obstructive coronary artery disease. The major drawback of coronary artery stenting is the occurrence of in-stent restenosis (ISR), which has been reported to occur in 11 to 46% at 6 months in bare metal stents (BMS) [2]. With introduction of drug eluting stents (DES), early ISR became less common and nowadays about 76% of revascularizations are performed using DES [3]. However, ISR still poses a major problem in coronary revascularization therapy with more than 200.000 estimated cases of DES ISR in the US alone. Late catchup in ISR when using DES has also been discussed [4, 5]. Moreover, in-stent thrombosis has been identified as a relevant problem in DES [6]. Another potential late complication of DES is the occurrence of stent fractures. The latter is considered a predisposing factor for ISR and late thrombosis. Coronary stent fractures are diagnosed in about 3% of patients [7], but autopsy data reports a much higher frequency of up to 29% [8].

While acute in-stent thrombosis typically becomes symptomatic with chest pain, the detection of ISR is more problematic as patients are often asymptomatic and about half of the patients with significant ISR do not experience any symptoms [9]. In addition, even complex noninvasive diagnostic tests such as myocardial single photon emission computed tomography (SPECT) yield only moderate results for detecting ISR [10, 11]. As a consequence, direct stent imaging appears to be worthwhile. Coronary catheter angiograms are costly and associated with a 0.1% mortality [12], whereas coronary magnetic resonance (MR) angiography after coronary stenting is still in an experimental stage [13]. Thus, coronary computed tomography (CT) angiography evolved as the only non-invasive diagnostic test allowing for direct visualization of coronary stents and, therefore, non-invasive detection of ISR, stent thrombosis and stent fractures. 

## 2. CT Imaging of Coronary Stents: The Past

The first report on localizing a coronary stent with unenhanced electron beam CT (EBCT) was published in 1995 [14]. Few groups generated a small amount of data on the use of EBCT for assessing coronary stent patency. Due to the limited spatial resolution of EBCT, direct visualization of the stent lumen was not possible and an indirect approach was applied to assess stent patency. For this purpose, contrast enhancement was determined distally to the stent and compared with the contrast enhancement pattern proximal to the stented segment, in the thoracic aorta or the left ventricle. Stent patency was assumed if the contrast enhancement distally to the stent matched the proximal coronary, aortic or left ventricular contrast enhancement pattern [15, 16]. Applying this technique, one has to be aware that contrast enhancement distal to any obstructed stent is influenced by retrograde filling via collateral vessels. Using this approach, a sensitivity of about 48–100% for detecting ISR or stent occlusion with a high negative predictive value of 80.5–100% was achieved (Table 1) [17]. For several reasons, including the inability to quantitatively assess the degree of ISR and its limited availability, EBCT imaging of coronary stents did not gain clinical acceptance and was soon pushed aside by multislice CT (MSCT).

With the simultaneous introduction of 4-slice CT scanners by all major vendors in 1998 and introduction of gating techniques for cardiac MSCT in 2000 [20], 4-slice CT became the first intensely used non-invasive imaging modality for assessing coronary artery stents. With its limited temporal and spatial resolution, direct visualization of the stent lumen was almost impossible and early studies focused on the visual assessment of the distal runoff [21]. This approach permitted the reliable detection of stent occlusion, but reliable assessment of ISR was not possible. Moreover, contrast enhancement distal to any stent is no absolute indicator of stent patency as retrograde filling via collaterals may also result in peripheral contrast enhancement. Dynamic assessment of coronary contrast enhancement, as it has been established for the EBCT assessment of coronary stents, was only sporadically reported [22].

Direct visualization of the stent lumen became feasible after 16-slice CT with improved temporal resolution and submillimeter spatial resolution was introduced in 2002. Only then, coronary CT angiography gained broader acceptance. With 16-slice CT direct assessment of the stent lumen became the primary goal of the examination in order to directly visualize ISR. The results from several studies on CT imaging of coronary stents were promising with sensitivities of 54% to 100% for detecting ISR (Table 2). Results were particularly promising after stenting of coronary artery bypass graft, where motion is markedly less and stents are bigger when compared with native coronary vessels [36]. However, on average, about 14% of stents were not evaluable with 16-slice CT [40] and even under ideal conditions in several phantom studies, only an average of 54% of the stent lumen were visible with CT [41, 42]. Gilard and coworkers showed that larger stents allowed for a better assessability of the stent lumen. Correspondingly, the sensitivity for detecting ISR increased from 54% in stents with a diameter of ≤3 mm to 86% in stents >3 mm [30]. These findings were also confirmed by data from phantom studies [43]. Using 16-slice CT technology, the basics for modern CT imaging of coronary stents including image acquisition, postprocessing, and data analysis were elaborated and the requirements for the rapid advancement of scanner hard- and software were identified.

## 3. Issues in CT Imaging or Coronary Stents

There are some specific technical issues in CT imaging of coronary stents. These include blooming artifacts due to beam hardening and partial volume effect, motion artifacts, geometric effects due to cardiac anatomy, and, last but not least, intravascular contrast enhancement.

Blooming is probably the most discussed issue in coronary stent imaging. It is mainly due to metal artifacts and the partial volume averaging effect. Blooming describes an effect where the stent struts appear to be thicker, causing an underestimation of the stent lumen. In fact, the presence of high-density objects such as the metal struts from stents or dense calcifications cause beam hardening, where lower energy photons of the X-ray beam are more rapidly absorbed, causing the beam to be more intense once it reaches the detector. Partial volume averaging also contributes to blooming artifacts. It is inherent with CT, as the CT number of each voxel represents the average attenuation of the materials within the voxel. In some situations, dark streaks, known as streak artifacts, may also be seen in the presence of metal. The latter are mostly due to a lack of attenuation data and an inaccurate beam hardening correction in filtered back projection. 

**Figure 1:**
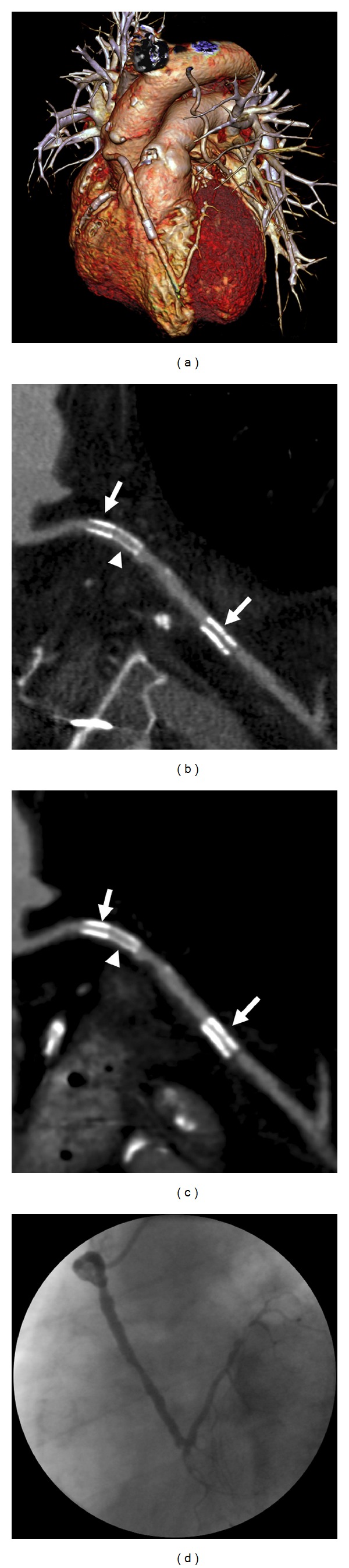
62-year-old male patient with a history of myocardial infarction and surgical revascularization therapy. 14 years after surgery, he developed CABG stenoses and subsequent stenting. CT was performed for ruling ISR. The 3D-volume rendered CT image shows the course of the stented vein graft to the LAD. A left internal mammary artery graft to a marginal branch is also depicted (a). Multiplanar curved reformats reconstructed with a dedicated sharp convolution kernel (b) and a smooth standard convolution kernel for cardiac CT angiography (c) show three CABG stents with a nominal diameter of 3 mm each. There are two TAXUS stents with 132 *μ*m strut thickness (arrows) and a Xience V stent with 81 *μ*m strut thickness (arrowhead). There is notably more blooming with the thicker struts and the stent lumen is better visible with the dedicated convolution kernel. ISR was ruled out by CT. This finding was confirmed by coronary angiography (d).

There are several approaches to solve these problems, with minimizing the amount of metal being the most obvious solution. Consequently, stents with thin struts and a low metal to surface ratio are thought to cause fewer artifacts. In contrast, blooming is more pronounced in the presence of overlapping stent placement or complex scenarios such as bifurcation lesions where Y-, V-, T-, or crush stenting techniques were applied. The presence of heavy calcifications in a stented segment further aggravates metal artifacts as it contributes to beam hardening. However, in clinical routine practice, these relationships are not that simple. In several clinical studies strut thickness had no significant effect on image quality [44, 45], although stents with a strut thickness of more than 100–140 *μ*m appear to be associated with poorer image quality [46, 47] (Figure 1). 

The type of stent is also known to affect the results. With the atomic number having a disproportionally high effect on attenuation, the stent material is essential, too. Generally speaking, a relatively low density of the metal as in magnesium or cobalt-chromium alloys appears to be advantageous [48]. Consequently stents or stent markers made from materials with high atomic numbers such as gold or tantalum cause markedly more artifacts when compared with stents made from stainless steel or alloys such as elgiloy and nitinol [42, 49]. 

Another technique for minimizing metal artifacts is the use of high kV imaging to avoid the photon starvation effect. However, this will result in an increased radiation exposure of the patient and should therefore be avoided whenever possible. As partial volume averaging contributes to blooming artifacts, the use of thin sections and a small field of view is recommendable. In fact, improvements in spatial resolution had probably the greatest effect on improving visibility of the stent lumen. This has been shown with experimental high resolution CT scanners [50, 51] as well as in phantom studies using clinical CT scanners [52]. Only recently, dual energy techniques including so-called monoenergetic imaging or iterative reconstruction techniques became available for coronary imaging, providing new approaches towards the reduction of metal artifacts [53, 54]. 

**Figure 2:**
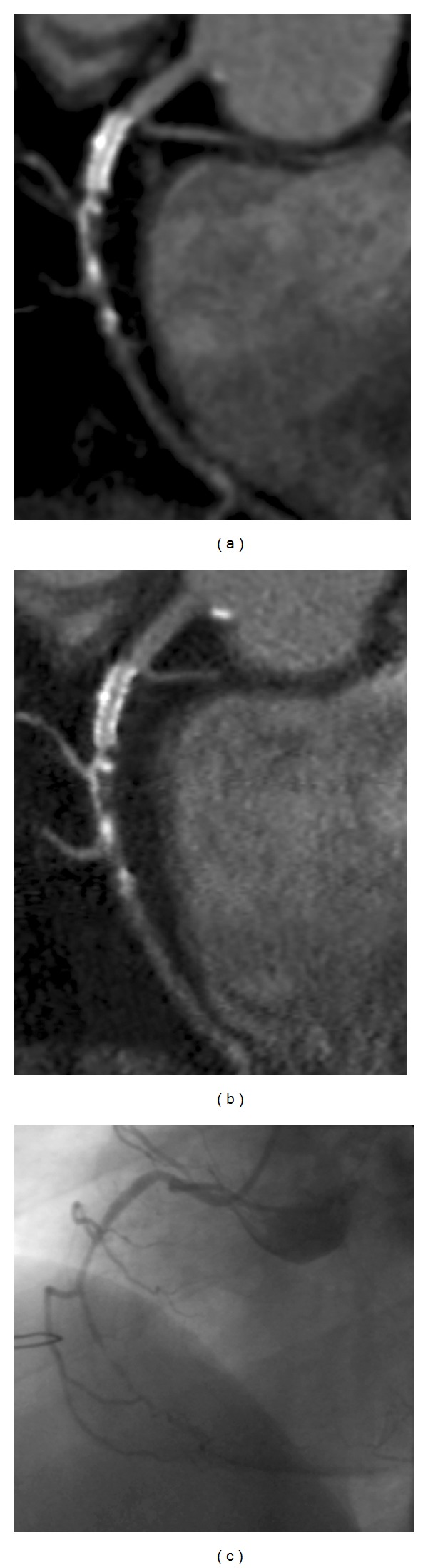
68-year-old male patient with a history of percutaneous coronary intervention with implantation of a 2.5 mm Xience V stent in the proximal RCA. CT shows the stent to be patent without relevant ISR, while there is a subtotal occlusion of the RCA distal to the stent (a, b). The finding was confirmed by coronary angiography (c). The stent lumen is better visible on images reconstructed with a dedicated convolution kernel (b), when compared with a standard convolution kernel (a). The use of a sharp convolution kernel goes along with a markedly increased image noise. Thus, the native vessel can be better assessed from images reconstructed with a smooth convolution kernel.

Interestingly, metal artifacts reduction algorithms as they were developed for CT imaging in the presence of metallic implants such as total hip replacement were never tested in cardiac CT. Instead, many vendors provide dedicated convolution kernels for image reconstruction. These (sharp) convolution kernels are designed to enhance the edges of high attenuation structures such as stent struts. Thereby, the blooming decreases at the costs of an increased image noise [55]. With current iterative reconstruction techniques a powerful tool for reducing image noise became available, compensating for the increased image noise [56]. The use of these dedicated reconstruction kernels is strongly recommended for assessing stent lumen, while the nonstented coronary artery segments should be assessed from image data reconstructed with a standard cardiac convolution kernel (Figure 2). 

Like in any type of coronary CT angiography motion artifacts either due to breathing or cardiac motion need to be overcome. With scan times below 10 s in 64-slice dual source CT (DSCT) scanners motion artifacts due to breathing does not pose a relevant problem anymore. Residual cardiac motion still poses a major problem. It causes blurring and particularly in high contrast objects such as coronary stents it disproportionally exacerbates the negative effects of blooming on image quality. Image quality and reliability of CT-value measurements inside stents are known to deteriorate with increasing heart rate [57]. Lowering the heart rate and improving temporal resolution are standard approaches towards this issue. In the particular setting of coronary stent imaging, however, improving the temporal resolution by means of multisegmental image reconstruction did not prove beneficial. Groen and coworkers even concluded that reduction in heart rate is more effective than improving the temporal resolution [58].

From several phantom studies, it is known that the angulation of the stent to the scan plane has a relevant effect on the visibility of the stent lumen [52, 59]. The lumen is described to be best visible if the stent was positioned 0° or 90° to the *z*-axis. However, except for the mid-section of the right coronary artery, the course of the coronary arteries is typically angulated. Thus, anatomy adds to the difficulties in CT imaging of coronary stents.

In addition to the scanner-related aspects, a sufficient intravascular contrast enhancement, ideally of more than 250 HU, is needed. This is a prerequisite for coronary CT angiography, but, in coronary stent imaging, a distinct contrast enhancement is of even more importance, as other factors such as image noise due to sharp convolution kernels or beam hardening artifacts in the presence of stents negatively affect contrast-to-noise ratios. Moreover, the selection of optimized windows settings, as described in Section 4, requires a good intravascular attenuation to permit delineation of vessel lumen, neointima inside the stent, and metal from the stent struts.

## 4. Considerations for Image Assessment

Coronary stent patency and ISR may be assessed in different ways. In the early days of coronary (4 slice), CT angiography direct visualization of the stent lumen was not possible. At that time, the intracoronary contrast enhancement distal to the stent was assessed as an indicator of stent patency. However, it is no absolute measure and may be false positive due to retrograde filling. Moreover, it does not provide information on the degree of ISR. A different approach uses dynamic scans as described for EBCT. The quantitative assessment of time-enhancement curves proximal and distal to a stent might be more reliable than mere visual assessment. This hypothesis, however, has not yet been validated.

With introduction of 16-slice CT scanners, more reliable approaches were sought. One of these techniques is the so-called pixel count method, where all pixels inside the stent lumen with a CT value above the lowest CT value proximal to the stent are counted in order to determine the presence of a stenosis. If more than 50% of the voxels inside a stent fulfilled this criterion, relevant ISR was assumed [32]. However, with a sensitivity and specificity of 75% and 88%, this method did not find its way into clinical routine practice. In another approach, the difference of the CT-values measured proximal and inside a stent were shown to be a good predictor of an at least 50% ISR, with a difference of 75 Hounsfield units (HU) being the most reliable threshold [38]. The most obvious technique, direct visualization of the stent lumen, proved to be the most reliable technique and was finally accepted as standard of practice. Although blooming still hampers this approach, it became accepted as is the most intuitive and easiest way. By using a wide window of ≥700 HU with a center of about 200 HU, there appears to be an acceptable tradeoff between blooming and visibility of the stent lumen. In addition, the CT values proximal and inside a stent are commonly measured [60]. However, one has to be aware that beam hardening usually causes a 60–100 HU overestimation of the CT-values inside a coronary stent. Therefore, measuring CT values is of limited value, while the visual assessment of attenuation differences, as they may be seen in stenotic lesions, are considered sufficiently reliable with 64-slice CT scanners.

## 5. CT Imaging of Coronary Stents: Current Status

In the first decade of CT imaging of coronary artery stents, lessons on the ideal scan protocol and image assessment were learned as described above. With introduction of 64-slice CT scanners, coronary CT angiography and concomitantly coronary stent imaging experienced its breakthrough in clinical routine practice. The increase in the number of slices from 4 to 64 went along with a decrease in section thickness from 1.25 mm to 0.5 mm and an increase in temporal resolution from about 250 ms to 83 ms or less.

Despite these marked improvements in scanner hardware, phantom studies still indicate relevant limitations of CT imaging of coronary artery stents with an artificial lumen narrowing in the range of 10% to 60% depending on the type of stent [48, 52]. With smaller stents, the artificial lumen narrowing is even more pronounced [61]. 

On first sight, these phantom studies still appear discouraging, but the clinical evidence tells a different story. Like conventional coronary, CT angiography for coronary artery disease, the application of 64-slice coronary CT angiography has a very high negative predictive value in range of 78–100% for exclusion of in-stent restenosis, while its positive predictive value is markedly worse (25–100%; Table 3). These results further improved with recent DSCT scanners (Table 4). Moreover, the number of stented segments, which had to be excluded from analysis progressively decreased from an average of 14% in 16-slice CT [62] to 8% with state-of-the-art scanners (Table 4). 

There are three meta-analyses on the value of 64-slice CT imaging in coronary artery stents [84–86]. The overall sensitivity, specificity, PPV, and NPV for assessable stents as reported by Kumbhani and coworkers were 91%, 91%, 68%, and 98%. If all stents were included in the analysis, the overall sensitivity, specificity, PPV, and NPV decreased to 87%, 84%, 53%, and 97%, respectively [85]. These results were much better when compared with earlier meta-analyses based on a mixture of 16- and 64-slice CT [62, 87], indicating the positive effect of improved spatial and temporal resolution on image quality. However, the interpretation of these current results is still controversial. Two of the meta-analyses on 64-slice CT are based on the identical set of clinical studies, but come to controversial conclusions. While Sun and Almutairi consider 64-slice CT as a reliable alternative to conventional coronary angiography [86], Kumbhani et al. conclude that stress imaging remains the most acceptable noninvasive technique for diagnosing ISR [85].

With 64-slice, CT blooming and motion artifacts due to heart rate variations including arrhythmias were the most common causes for impaired image quality. In addition, stent-related factors such as stent diameter, strut thickness, stent design, and type of stent placement (e.g., overlapping stenting) were shown to influence the visibility of coronary stent lumen. There is a consensus that stents with a diameter below 3 mm are more likely to be inaccessible than stents with a diameter of 3 mm or more [60, 66, 72, 88]. At large, thick stent struts are more likely to go along with an inaccessible stent lumen. However, there is no generally accepted definition of thin or thick struts and different thresholds have been used in the literature [71, 89]. In addition, more complex procedures with bifurcation or overlapping stenting, where there are multiple layers of metal cause more blooming, thereby limiting the visibility of the stent lumen [64, 88]. The effect of the stent design remains unclear as no differences were found between open and closed cell design [47, 68]. 

## 6. CT Imaging of Coronary Stents Beyond ISR

Most non-invasive imaging strategies in the presence of coronary stents focus on ISR as it is often asymptomatic, despite hemodynamic relevance of a stenosis. In contrast, in-stent thrombosis typically goes along with chest pain and requires acute therapy. Correspondingly, there is almost no data on the diagnostic value of cardiac CT in in-stent thrombosis. In an initial series including 79 patient with acute onset of chest pain, the sensitivity, specificity, and positive, and negative predictive values of 64-slice CT for the detection of in-stent thrombosis were 95%, 93%, 83%, and 98%, respectively [94]. When considering these data, one has to be aware that this setting is not an appropriate indication for cardiac CT [95].

**Figure 3:**
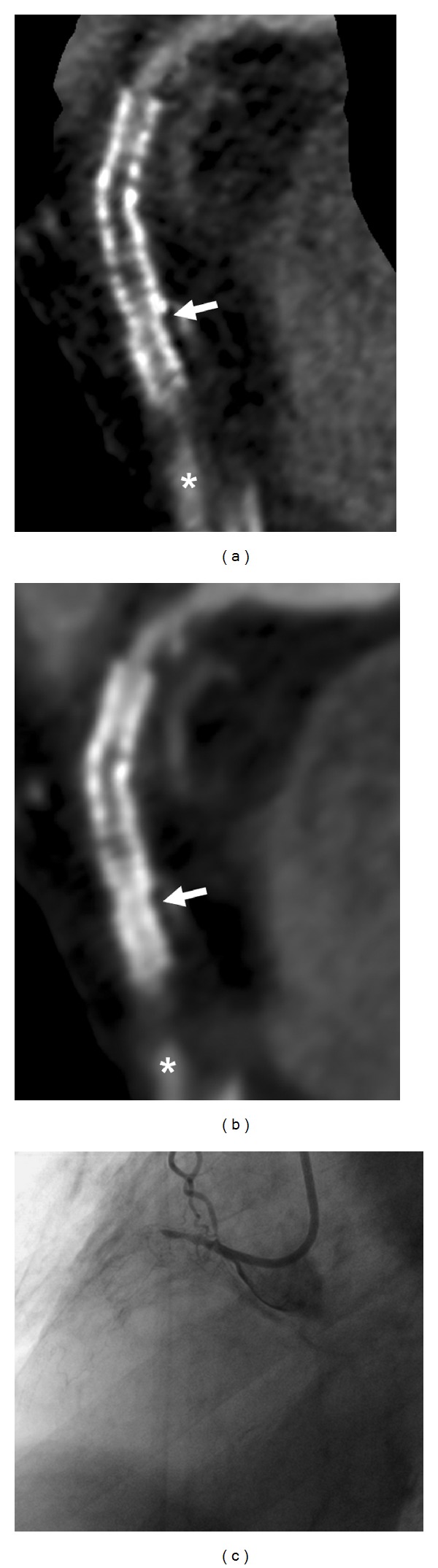
73-year-old male patient with a history of myocardial infarction and percutaneous recanalization of the RCA with implantation of a 3 mm Coroflex blue and 3 mm Vision stent. CT images computed with a dedicated convolution kernel (a) and a smooth kernel (b) show total stent occlusion with distal filling of the vessel (asterisk) via collateral flow. This finding was confirmed by conventional coronary angiography (c). CT also showed a step with incongruent course of the stents, indicating stent fracture.

Stent fractures are a completely different issue. Considering the discrepancy between 3% clinically suspected stent fractures and a reported occurrence of up to 29% in autopsy series, new diagnostic strategies are needed to deal with this issue [7, 8]. This is of particular relevance as stent fractures are thought to be a predisposing factor for ISR and in-stent thrombosis [96] (Figure 3). So far, there is only scarce data on this topic. Data from a phantom study indicates that 64-slice CT is more accurate than conventional cineangiography for detecting coronary stent fractures with an overall accuracy of 84.1% for CT versus 73.9% for fluoroscopy [97]. This has also been confirmed in the only patient series dealing with stent fractures [98]. A study by Hecht et al. focused on the detection of stent gaps by means of coronary CT angiography. The latter either represent stent fracture or overlap failure. CT has been shown to be markedly more sensitive for detecting gaps between multiple stents, when compared with fluoroscopy (16.9% versus 1.0%) [99]. Considering the currently available data, cardiac CT appears to be better suited than conventional coronary angiography for detecting stent fractures and cardiac CT might be the method of choice for detecting coronary stent fractures. While stent gaps were shown to be associated with ISR [99], the clinical relevance of these findings has still to be determined.

## 7. Discussion

CT imaging of coronary stents rapidly evolved from a scientific toy to a clinical tool. This development is reflected by its consideration in the current guidelines on coronary CT angiography. While in the 2006 American Heart Association (AHA) scientific statement on cardiac computed tomography CT imaging of stents was generally discouraged [100], it is now considered appropriate in some indications such as for risk assessment after revascularization in asymptomatic patients with a history of left main coronary artery stenting and a stent diameter of equal or more than 3 mm. While it is still considered inappropriate in stents smaller than 3 mm, its value in symptomatic patients is unknown [95]. Accordingly, the 2010 expert consensus on the use of cardiac CT stated “Thus, in a patient known to have larger stents and whose clinical presentation suggests low-to-intermediate probability for restenosis, 64-channel coronary CTA may be a reasonable alternative to invasive angiography to rule out significant in-stent restenosis” [101].

These recommendations reflect the evidence on 64-slice cardiac CT. With DSCT and up to 320-slice single source CT scanners, further achievements were made. The significance of these improvements is likely to be valued in updated guidelines. As CT is quick and non-invasive, it is usually preferred by patients over invasive or lengthy procedures such as catheter angiography or MR imaging. Moreover, it is cheaper and requires almost no preparation time. However, there are some drawbacks including the patient's exposure to contrast material and radiation. Only recently, several investigators compared prospectively ECG-triggered sequential and retrospectively ECG-gated spiral scanning. While there were no relevant differences in stent assessment, this technique allowed for cutting down the radiation exposure by 75%. With 2.2–5.7 mSv, it is in the range of the annual exposure to background radiation [76, 81, 90]. Another shortcoming is the fact that these encouraging results do not apply for all types and sizes of coronary stents as shown above. Nevertheless, coronary CT angiography provides better results for detecting ISR than any other non-invasive diagnostic test including myocardial SPECT [10, 11]. 

## 8. Future Perspectives

Several current developments will further improve coronary stent imaging by means of cardiac CT. Most of these are incremental improvements of scanner hardware such as a further improvement of temporal resolution, which is currently in the range of 75 ms. The continuous improvement in spatial resolution will help to reduce blooming due to the partial volume effect. State-of-the-art CT scanners now have a collimated slice thickness of 0.5 mm and a spatial resolution down to 0.2 mm has been shown to be beneficial for coronary stent imaging [50, 51]. Most recent DSCT scanners permit so-called high pitch scanning, allowing for a dose reduction below 2 mSv [102]. This technique also works in the presence of coronary stents [103], but so far there is no patient data with this technique. 

New imaging concepts which combine morphological and functional aspects are the most exciting development. Only recently, CT perfusion imaging became feasible, giving way for new examination strategies, which combine CT angiography and dynamic perfusion imaging for assessing the functional relevance of morphological findings. These features can both be integrated in a single comprehensive CT examination. Initial patient data indicates the effectiveness of this imaging strategy [104]. Alternatively modern hybrid imaging techniques such as PET/CT or SPECT/CT with integrated 64-slice CT scanners permit the combination of morphologic and metabolic imaging. However, these imaging modalities were designed for technically less demanding tasks such as oncologic imaging. Consequently, the CT component of these hybrid modalities usually limps behind the current developments in cardiac CT imaging. Thus, comprehensive single modality examination strategies including perfusion imaging and state-of-the-art morphological imaging are more appealing.

Not only imaging technique is improving, stents theirselves are also changing. While drug eluting stents made from metal are the current mainstay in coronary revascularization therapy, drug eluting biodegradable stents are under clinical evaluation [105]. Naturally, these stents are made of less dense materials with lower atomic numbers, particularly if biodegradable scaffolds are used. These stents will be almost CT transparent, therefore permitting almost unrestricted CT imaging of the stent lumen.

## 9. Conclusion

Coronary CT imaging of coronary artery stents evolved as a reliable tool in the diagnostic workup of patients after coronary revascularization therapy. With 64 slice or newer generation, CT scanners cardiac CT is well suited to rule out ISR in the presence of coronary stents with a diameter equal to or exceeding 3 mm. In these patients, cardiac CT has to be considered in clinical pathways as an alternative to invasive coronary angiography for the workup of patients with suspected ISR after revascularization. The development and evaluation of comprehensive examination protocols assessing morphology and hemodynamic significance of potential ISR will further enhance the diagnostic potential of cardiac CT after coronary stenting. 

## Figures and Tables

**Table 1:** Summary of studies on EBCT imaging for assessing coronary stent patency.

Author/year	Patients (*n*)	Stents (*n*)	Nonevaluable (%)	Sensitivity (%)	Specificity (%)	PPV (%)	NPV (%)
Schmermund et al. 1996 [15]	22	na	9	100	100	100	100
Pump et al. 1998 [17]	177	285	7.2	82.3	97.6	77.8	98.2
Pump et al. 2000 [18]	202	321	4	78	98	82	97
Knollmann et al. 2004 [16]	117	152*	9.3	48	90.1	67.7	80.5
Zhou et al. 2005 [19]	25	35	8	85	92.9	75	96.5

Total/mean	543	793	7.5	78.7	95.7	80.5	94.4

na: not available; PPV: positive predictive value; NPV: negative predictive value; *stented segments.

**Table 2:** Summary of studies on 4-, 16-, and 40-slice CT imaging for assessing coronary stent patency and rule out of ISR.

Author/year	Scanner (rows)	Patients (*n*)	Stents (*n*)	Non evaluable (%)	Sensitivity (%)	Specificity (%)	PPV (%)	NPV (%)
Maintz et al. 2003 [23]	4	29	47	19.1	100	100	100	100
Krüger et al. 2003 [21]	4	20	32	0	100	100	100	100
Ligabue et al. 2004 [24]	4	48	72	17.8	100	100	100	100
Mazzarotto et al. 2006 [25]	4	24	34	9.4	91.3	66.6	95.5	50
Schuijf et al. 2004 [26]	16	22	68	23	75	96	na	na
Gilard et al. 2005 [27]	16	29	29	7	100	93	100	93
Cademartiri et al. 2005 [28]	16	51	74	na	83.3	98.5	83.3	97.3
Watanabe et al. 2006 [29]	16	31	42	16.7	83	90	63	96
Gilard et al. 2006 [30]	16	143	128 (≤3 mm)	45.7	54	100	100	94
			104 (<3 mm)		86	100	100	99
Kitagawa et al. 2006 [31]	16	42	61	31.1	100^(1)^	100^(1)^	100^(1)^	100^(1)^
Ohnuki et al. 2006 [32]	16	16	20	5	75	88	75	93
Kefer et al. 2007 [33]	16	50	73	na	67	98	92	89
Chabbert et al. 2007 [34]	16	134	145	8.3	90.5^(2)^	66.5^(2)^	42^(2)^	96^(2)^
Soon et al. 2007 [35]	16	37	47	4	71	97	83	95
Mühlenbruch et al. 2007 [36]^(3)^	16	14	20	na	100	100	100	100
Tedeschi et al. 2008 [37]	16	72	90	21	82	96	87	94
Kitagawa et al. 2008 [38]	16	38	47	26	100	74	67	100
Gaspar et al. 2005 [39]	40	65	111	4.5	74.1	83.3	58.8	90.9

Total/mean		264	865	18.1	82.0	90.7	83.6	93.9

na: not available; PPV: positive predictive value; NPV: negative predictive value; ^(1)^subgroup of 21 assessable stents with angiographic correlation; ^(2)^mean of two observers; ^(3)^only CABG stents.

**Table 3:** Summary of studies on 64-slice CT angiography for assessing coronary stents.

Author/year	Patients (*n*)	Stents (*n*)	Non evaluable (%)	Sensitivity (%)	Specificity (%)	PPV (%)	NPV (%)
Rixe et al. 2006 [63]	64	102	42	86	98	86	98
Van Mieghem et al. 2006 [64]	70	162	na	100	91	67	100
Rist et al. 2006 [65]	25	46	2	75	92	67	94
Oncel et al. 2007 [66]	30	39	0	89	95	94	90
Cademartiri et al. 2007 [67]	182	192	7	95	93	63	99
Ehara et al. 2007 [68]	81	163	12	92	81	54	98
Carrabba et al. 2007 [69]	41	87	0	84	97	92	97
Das et al. 2007 [70]	53	110	2.7	97	88	77	98
Schuijf et al. 2007 [71]	50	76	14	100	100	100	100
Carbone et al. 2008 [72]	41	74	19.5	75	86	71	89
Manghat et al. 2008 [73]	40	114	9.6	85	86	71	89
Hecht et al. 2008 [74]	67	132	0	94	74	39	99
Nakamura et al. 2008 [75]	49	75	14.6	67	92	29	98
Andreini et al. 2009 [47]	100	179	5	87	98	92	96
Pontone et al. 2009 [76]	80 (gating)	48	8	92	94	85	87
	80 (triggering)	66	6	73	96	80	94
Haraldsdottir et al. 2010 [77]	93	140	14	27	95	67	78
Papini et al. 2010 [78]	26	42	20	97	100	97	100
Abdelkarim et al. 2010 [79]	55	122	13.2	91	95	96	91
Chung et al. 2010 [44]	60	91	24.2	90	74	58	95
Wykrzykowska et al. 2010 [80]	52	75	36	33.3	91.7	57.1	80.5
Andreini et al. 2011 [81]	85 (gating)	163	5	86	97	91	96
	83 (triggering)	174	7	94	100	100	98
Zhao et al. 2011 [82]	18	29	0	100	95	89	100
Zhang et al. 2012 [83]	83	171	28.7	100	69	25	100

Total/mean	1608	2672	12.1	84.4	91.1	73.9	94.6

na: not available; PPV: positive predictive value; NPV: negative predictive value.

**Table 4:** Summary of studies on 64-slice DSCT and 320-slice CT for assessing coronary stents.

Author/year	Patients (*n*)	Stents (*n*)	Scanner type	Non evaluable (%)	Sensitivity (%)	Specificity (%)	PPV (%)	NPV (%)
Oncel et al. 2008 [60]	35	48	64 DSCT	15	100	94	89	100
Pugliese et al. 2008 [88]	100	178	64 DSCT	5	94	92	77	98
Pflederer et al. 2009 [89]	112	150	64 DSCT	10	84	95	73	97
Zhao et al. 2011 [90]	30 (gating)	56	64 DSCT	12.5	94	87	77	97
	30 (triggering)	59	64 DSCT	13.6	100	84	68	100
Veselka et al. 2011 [91]	34	34	64 DSCT	0	100	74	18	100
Zhang et al. 2012 [92]	50	115	64 DSCT	0	69.2	91.2	50	95.9
De Graaf et al. 2010 [93]	53	89	320 MSCT	8	92	83	46	98

Total/mean	444	729		8	94.9	87.0	62.3	98.6

PPV: positive predictive value; NPV: negative predictive value.
